# Proteinuria is a clinical characteristic of intrahepatic cholestasis of pregnancy but it is not a marker of severity: A retrospective cohort study

**DOI:** 10.1371/journal.pone.0310217

**Published:** 2024-09-11

**Authors:** Hadel Watad, Aviran Ohayon, Raanan Meyer, Adiel Cohen, Eran Kassif, Michal Fisher-Bartal, Rakefet Yoeli, Shali Mazaki-Tovi

**Affiliations:** 1 Department of Obstetrics and Gynecology, Chaim Sheba Medical Center, Tel-Hashomer, Ramat-Gan, Israel; 2 Faculty of Medicine, Tel Aviv University, Tel Aviv, Israel; 3 Department of Obstetrics, Division of Maternal-Fetal Medicine, Gynecology and Reproductive Sciences, McGovern Medical School, The University of Texas Health Science Center at Houston, Houston, TX, United States of America; Ann and Robert H Lurie Children’s Hospital of Chicago / Northwestern University Feinberg School of Medicine, UNITED STATES OF AMERICA

## Abstract

**Objectives:**

To determine the prevalence of proteinuria in patients diagnosed with intrahepatic cholestasis of pregnancy (IHCP), and the association between the presence of proteinuria and adverse pregnancy outcomes.

**Methods:**

This was a retrospective cohort study. The study included all pregnant patients between July 2014 and January 2022, at gestational age > 24weeks who had been diagnosed with IHCP and had completed a 24-hour protein collection. High order multifetal gestations were excluded. Patients were divided into 3 groups:1. IHCP without proteinuria (Non-proteinuric group);2. IHCP with proteinuria and normal blood pressure (Isolated proteinuria group), and 3. IHCP with proteinuria and elevated blood pressure (IHCP with preeclampsia (PET)). Primary outcome was defined as a composite maternal-fetal outcome including: preterm labor <34 weeks, arterial cord blood ph<7.1, rate of Cesarean delivery due to non-reassuring fetal monitoring. Parametric and non-parametric statistical methods were used for analysis.

**Results:**

A total of 272 met all inclusion criteria and were included, 94 patients (34.5%) had proteinuria; of them, 67 (24.6%) had isolated proteinuria and 27 (9.9%) had PET. Demographic parameters were comparable among the groups. Patients with PET had higher rates of in-vitro fertilization (IVF) treatments, twin gestation and elevated serum creatinine and urea levels. The rate of composite adverse pregnancy outcome was higher in patients with PET compared with patients with and without proteinuria (14/27 (51.9%) vs. 18/67 (26.9%) vs. 49/178 (27.5%), respectively, p = 0.03).

**Conclusions:**

Approximately 35% of patients with IHCP have proteinuria. The presence of PET, rather than isolated proteinuria, is associated with adverse pregnancy outcome.

## Introduction

Intra-hepatic cholestasis of pregnancy (IHCP) is a common liver disease [1], with a prevalence of up to 4% of pregnancies [2]. Patients commonly present in the third trimester with pruritus accompanied with elevated serum liver tests and serum bile acids [3–7].

IHCP primarily affects the fetus and resolves shortly after delivery [8,9]. However, accumulating evidence shows that while IHCP is associated with adverse pregnancy outcomes including preeclampsia (PET) [10–12], abnormal lipid profile [13] and glucose intolerance [13,14], preterm labor (PTL), meconium and fetal death (FD) [1,15], it might have lifelong consequences including increased risk of later hepatobiliary cancer and immune-mediated and cardiovascular diseases [16].

IHCP and PET share multiple characteristics: similar risk factors, biochemical changes, and adverse pregnancy outcomes. This overlap poses a clinical diagnostic dilemma in some cases which prompt clinicians to include PET in the differential diagnosis and to order a 24-hour protein collection test. This practice is based on the premise that proteinuria is a unique feature of PET [17], but not of IHCP. Moreover the findings that women with gestational proteinuria have angiogenic factors that are intermediate between those of normal pregnancies and pre-eclampsia [18], promote consideration that these women have an early form of pre-eclampsia (ISSHP guidelines for the classification, diagnosis and management of the hypertensive disorders of pregnancy [19]). Nevertheless, there is no evidence that supports this assumption.

The rationale that proteinuria might be a clinical characteristic of IHCP is based on several findings. Chronic cholestatic liver diseases are associated with tubulointerstitial nephropathies. Moreover, the term " Cholemic nephropathy " describes an association between obstructive cholestasis and renal dysfunction [20–22]. Several pathogenetic mechanisms had been suggested, including: 1. oxidative stress induced by renally eliminated cholephiles [23] and bile acid-induced oxidative stress, leading to tubular damage; 2. portal and systemic endotoxemia via increased translocation from the intestine due to lack of enteral bile acids or alternative cholephiles; 3. increased production and/or expression of vasoactive mediators; 4. direct tubular effects of bile, and 5. volume depletion [20,22–36].

Collectively, these findings raise the question whether proteinuria might be a clinical characteristic of IHCP. The question is of special importance as proteinuria is considered a unique feature of PET and has not been described in any other “great obstetrical syndromes” [37] or even in less frequent obstetrical complications.

Our hypothesis is that IHCP associated with proteinuria and adverse pregnancy outcomes. Therefore, the aims of this study were to determine the prevalence of proteinuria in patients diagnosed with IHCP and the association between proteinuria and adverse pregnancy outcome in patients with IHCP.

## Methods

A retrospective cohort study between July 2014 and January 2022 at a single tertiary referral center. The study included all pregnant patients at gestational age > 24 weeks who were diagnosed with IHCP and completed a 24-hour protein urine collection. Cases of IHCP were identified by searching the electronic database using the words "Intra-hepatic cholestasis of pregnancy", "cholestasis of pregnancy", "elevated liver enzyme", "pruritis". IHCP was defined as new onset, persistent pruritus in combination with elevated bile acids in the absence of other identifiable liver disorder. Total bile acids (TBA) were determined after 8 hours fasting and were considered elevated if the levels were ≥ 10 μmol/L [38–40]; in case of repeated test during current pregnancy, the maximal levels were taken. Abnormal liver enzymes were defined as 1 or more abnormalities in alanine aminotransferase (ALT) and/or aspartate aminotransferase (AST). Proteinuria was defined as 24-hour protein urine collection above 300 mg/dl [41]. We included singletons and twins pregnancies only. Any higher order multifetal pregnancies were excluded due to a small number of cases and lack of homogeneity. Patients with baseline proteinuria (pre-pregnancy), primary hepatic and/or renal dysfunction were excluded. Patients with incomplete data and patients who did not complete a urine collection test were also excluded.

Demographic and clinical data including maternal age, body mass index (BMI), smoking status, obstetric history, gestational diabetes mellitus, gestational age at diagnosis of IHCP, laboratory workup, preterm pre mature rupture of membranes (PPROM), presence of meconial amniotic fluids at delivery, gestational age at delivery, PTL, mode of delivery, fetal weight and percentile (using population-specific curves [42]), fetal and neonatal death, Apgar score at 1 and 5 minutes, Neonatal Intensive Care Unit (NICU) admission and umbilical pH were collected from the electronic database.

Our practice is to hospitalize for evaluation all patients with suspected IHCP <37 weeks. Workup evaluation includes: laboratory tests including complete blood count (CBC), coagulation tests, renal functions test, liver enzymes and TBA twice a week; vital sign measurements, fetal monitoring by cardiotocography (CTG) three times per day; estimated fetal growth assessment once in 10–14 days; and 24-hour protein urine collection test is performed for all hospitalized patients with suspected IHCP. Once the diagnosis is confirmed, treatment with Ursodeoxycholic Acid is initiated.

IHCP severity was defined according to TBA levels as severe (>40 μmol/L), moderate (20–40 μmol/L), or mild (10–20 μmol/L) [43–45]. Cases with mild cholestasis and reassuring fetal tests are discharged and outpatient surveillance with twice weekly fetal monitoring is recommended. Cases with moderate and severe IHCP are hospitalized until labor induction.

Cases with mild or moderate cholestasis TBA (> 10 μmol/l and <40 μmol/l) are induced at 37 weeks of gestation, cases with severe cholestasis (TBA 40–99 μmol/l) are induced at 36 weeks of gestation, and cases with TBA≥ 100 μmol/l are induced at 34–36 weeks of gestation [46].

PET was defined as blood pressure values of ≥140/90 mm Hg accompanied by proteinuria of ≥0.3-g protein in a 24-hour urine specimen first diagnosed at >20 weeks of gestation [41]. Severe PET was defined as PET accompanied by one of the following: blood pressure values of ≥160/110 mm Hg on 2 occasions that were at least 6 hours apart while on bed rest, proteinuria of ≥5 g in a 24-hour urine collection or ≥3‏ + on 2 random urine samples that were collected at least 4 hours apart, oliguria of <500 mL in 24 hours, cerebral or visual disturbances, pulmonary edema or cyanosis, epigastric or right upper-quadrant pain, impaired liver function, thrombocytopenia, or fetal growth restriction [41].

Patients with IHCP were divided to 3 groups: 1. IHCP without proteinuria (The non-proteinuric group), 2. IHCP with proteinuria and normal blood pressure (Isolated proteinuria group), and 3. IHCP with proteinuria and elevated blood pressure (IHCP with PET). Pregnancy outcomes were compared among groups. Our primary outcome was defined as a composite outcome including the occurrence of any of the following: PTL <34 weeks, cord blood pH <7.1, cesarean delivery due to non-reassuring fetal monitoring. The manuscript was written and edited according to the STROBE statement.

Normality of continuous data was tested using the Kolmogorov–Smirnov test. Data are presented as percent and numbers or median and inter-quartile range (IQR), as appropriate. Kruskal–Wallis tests with *post-hoc* analysis by Mann–Whitney U-tests were used for comparisons of continuous variables among the different groups. Comparison of proportions was performed by Chi-square or Fisher’s exact tests. The p value of significance was 0.05. Statistical analyses were conducted using the IBM Statistical Package for the Social Sciences (IBM SPSS v.23; IBM Corporation Inc., Armonk, NY, USA).

Ethics statement: This study was conducted as a component of a broader research project, which has received ethical approval from Institutional Review Board on March 6^th^, 2022. Approval number: 9245-22-SMC. A written approval was obtained. Need for participants consent was waived by the ethics committee in this retrospective study due to the nature of data collection, which involved the analysis of pre-existing and de-identified records, and as such, direct interaction with the subjects did not occur. The main researcher had access to individual patients’ data during data collection. Data were accessed for this research on May 18, 2022.

## Results

**Fig 1:**
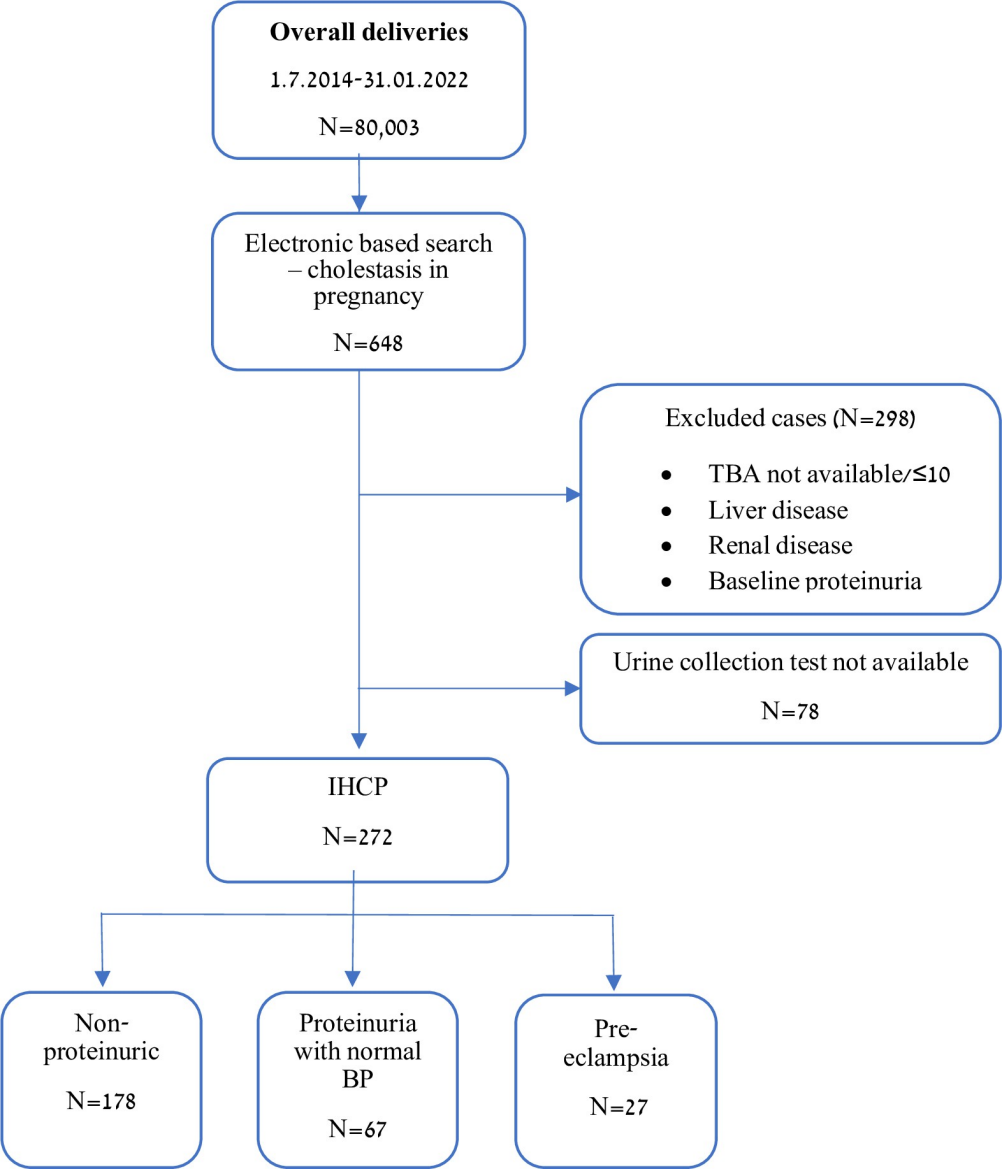
Flowchart study design. TBA-Total bile acids, IHCP- intra hepatic cholestasis of pregnancy, BP-Blood pressure.

Of the 80,003 deliveries during the study period, 648 (0.82%) were diagnosed with IHCP, 42% (272 patients) met the inclusion criteria of IHCP and were included in our study. Overall, 178 patients had IHCP without proteinuria (The non-proteinuric group). Ninety-four (34.5%) patients had proteinuria: 23.6% (67/272) had isolated proteinuria without elevated blood pressure (Isolated proteinuria group), while 9.9% (27/272) patients had combined proteinuria and elevated blood pressure and were diagnosed with PET (Fig 1).

Demographic and baseline clinical characteristics are presented in Table 1. Maternal age, smoking status, chronic comorbidities, pre-pregnancy BMI, gravidity, parity, history of PTL and cholestasis, were all comparable among the groups.

**Table 1:** Baseline demographics and clinical characteristics of study groups.

	Non-proteinuric (n = 178)	Proteinuric with normal BP (n = 67)	Preeclampsia (n = 27)	*p-*value
**Maternal age (years)**	31 (26–35)	32 (28–36)	33 (29–36)	0.139
**Smoker**	3 (1.7)	2 (3)	0 (0)	0.601
**Chronic hypertension**	1 (0.6)	0 (0)	1 (3.7)	0.147
**Pre-pregnancy BMI (Kg/m2)**	21.9 (20.0–24.9)	22.5 (21.2–25.7)	23.8 (19.7–28.8)	0.141
**Pre-gestational diabetes**	2 (1.1)	2 (3)	2 (7.4)	0.103
**Hypothyroidism**	14 (7.9)	6 (9)	2 (7.4)	0.953
**Thrombophilia**	5 (2.8)	3 (4.5)	2 (7.4)	0.458
**Gravidity**	2 (1–3)	2 (1–4)	2 (1–3)	0.114
**Parity**	1 (0–1.25)	1 (0–2)	0 (0–1)	0.057
**Previous preterm birth**	34 (19.1)	17 (25.4)	2 (7.4)	0.135
**IHCP in previous pregnancy**	43 (24.2)	13 (19.4)	2 (7.4)	0.289
**PET in previous pregnancy**	7 (3.9)	1 (1.5)	0 (0)	0.382

^a^ Data are presented as median and interquartile range (IQR) or N (%).

BP- Blood pressure, IHCP–intrahepatic Cholestasis of pregnancy, PET- preeclampsia.

Higher rate of conception via in-vitro fertilization (IVF) was found in the PET group compared with patients in the non-proteinuric group and the isolated proteinuria group (37% vs. 15.7% vs 25.4%, respectively, p = 0.018), and a higher percentage of twin gestations was also found in the PET group compared with the other two groups (Table 2).

**Table 2:** Clinical characteristics and laboratory data of index pregnancy.

	Non-proteinuric (n = 178)	Proteinuric with normal BP (n = 67)	Preeclampsia (n = 27)	*p*
**Spontaneous pregnancy** ^**c**^	142 (79.8)	49 (73.1)	15 (55.6)	**0.02**
**IVF pregnancy** ^**c**^	28 (15.7)	17 (25.4)	10 (37)	**0.018**
**Aspirin use during pregnancy**	5 (2.8)	3 (4.5)	1 (3.7)	0.803
**Twins**	38 (21.3)	21 (31.3)	11 (40.7)	**0.048**
**GCT (mg/dL)**	122 (97.7–144.)	122.5 (110.2–148.7)	123 (106–138.2)	0.639
**Diabetes**	40 (22.5)	15 (22.4)	6 (22.2)	1
**GA at pruritus onset (weeks)**	32 (29.2–34)	32 (30–34)	31 (29–34)	0.805
**GA at admission** **(weeks)**	34 (31–35)	33 (32–35)	32 (30–34)	0.163
**Maximal levels- serum TBA (μmol/L)**	26 (16–47.2)	24 (14–45)	33 (17–70)	0.304
**TBA >40 (μmol/L)**	59 (33.1)	22 (32.8)	11 (40.7)	0.725
**Maximal serum levels of AST (U/L)**	95 (52.7–161.)	114 (69–185)	81 (47–123)	0.17
**AST >40 U/L**	152 (85.4)	58 (86.6)	23 (85.2)	0.97
**Maximal serum levels of ALT (U/L)**	132 (69–242)	163 (95–311)	98 (52–190)	0.151
**ALT > 45 U/L**	151 (84.8)	57 (85.1)	22 (81.5)	0.896
**Minmial platelet levels (100/L)**	169.5 (131–206.25)	180 (156–221)	154 (130–208)	0.078
**Maximal serum creatinine levels (mg/dL)** ^**c**^^,^^**d**^	0.55 (0.4–0.6)	0.57 (0.53–0.7)	0.66 (0.58–0.87)	**0.001**
**Maximal serum urea levels (mg/dL)** ^**c**^^,^^**d**^	21 (17–25)	23 (19–27)	26 (22–37)	**0.001**
**Maximal protein levels at 24 H urine collection (mg/day)** ^**b**^^,^^**c**^	190 (140–240)	420 (360–600)	490 (360–1080)	**<0.001**
**GA at BP elevation (weeks)**	35.5 (34–36)	N/A	34 (31.5–35)	**0.013**
**Systolic BP at admission (mmgh)** ^**c**^^,^^**d**^	114 (107–120)	113 (106–120)	127 (116–135)	**<0.001**
**Diastolic BP at admission (mmgh)** ^**c**^^,^^**d**^	74 (68–80)	74 (69–79)	83 (77–87)	**<0.001**
**Maximal systolic BP (mmgh)** ^**c**^^,^^**d**^	126 (120–133.25)	127 (120–132)	147 (143–167)	**<0.001**
**Maximal diastolicBP (mmgh)** ^**c**^^,^^**d**^	79 (73–84)	79 (74–84)	90 (87–101)	**<0.001**
**Systolic BP >140 mmgh**	24 (13.5)	N/A	23 (85.2)	**<0.001**
**Diastolic BP >90 mmgh**	15 (8.4)	N/A	17 (63)	**<0.001**
**Systolic BP >160 mmgh**	3 (1.7)	N/A	8 (29.6)	**<0.001**
**Diastolic BP >110 mmgh**	0 (0)	N/A	5 (18.5)	**<0.001**

^a^ Data are presented as median and interquartile range (IQR) or N(%).

BP- Blood pressure, IVF-in vitro fertilization, GCT- Glucose challenge test, TBA-total bile acids, AST-Aspartate aminotransferase, ALT-Alanine transaminase, UDCA- Ursodeoxycholic acid, GA–gestational age.

b—P value < 0.05 in a comparison between non-proteinuric and Proteinuric with normal BP.

c- P value < 0.05 in a comparison between non-proteinuric and pre-eclampsia.

d- P value < 0.05 in a comparison between proteinuric with normal BP and pre-eclampsia.

Parameters that may be related to IHCP severity, including gestational age at diagnosis of cholestasis, gestational age at admission and maximal levels of serum TBA, AST and ALT were all comparable among three groups, except of a higher median level of serum creatinine and urea in the PET group compared with the other two groups (Table 3).

**Table 3:** Delivery data of index pregnancy.

	Non-proteinuric (n = 178)	Proteinuric with normal BP (n = 67)	Preeclampsia (n = 27)	*p-*value
**Gestational age at delivery** **(weeks)** ^**b**^^,^^**c**^^,^^**d**^	36 (35–37)	36 (35–36)	35 (33–36)	**0.001**
**BMI at delivery (Kg/m2)** ^**b**^^,^^**c**^	25.8 (23.4–28.7)	27.4 (24.8–30.1)	28.3 (26.2–31.0)	**0.05**
**PPROM**	10 (5.6)	1 (1.5)	1 (3.7)	0.368
**PTL < 34 weeks** ^**b**^^,^^**d**^	20 (11.2)	2 (3)	7 (25.9)	**0.004**
**Induction of labor**	114 (64)	34 (50.7)	15 (55.6)	0.148
**Mode of delivery**				
**Spontaneous vaginal delivery**	112 (62.9)	37 (55.2)	11 (40.7)	0.073
**Assisted vaginal delivery**	12 (6.7)	1 (1.5)	0 (0)	0.108
**Elective Cesarean section**	21 (11.8)	14 (20.9)	5 (18.5)	0.169
**Urgent Cesarean section** ^**c**^	34 (19.1)	14 (20.9)	11 (40.7)	**0.039**
**Meconium passage**	28 (15.7)	10 (14.9)	5 (18.5)	0.872

a- Data are presented as median and interquartile range (IQR) or N(%).

BP- Blood pressure, BMI-Body mass index, PPROM-Preterm premature rupture of the membranes, PTL-preterm labor.

b—P value < 0.05 in a comparison between non-proteinuric and Proteinuric with normal BP.

c- P value < 0.05 in a comparison between non-proteinuric and pre-eclampsia.

d- P value < 0.05 in a comparison between proteinuric with normal BP and pre-eclampsia.

The median of gestational age at delivery for pregnant women in the non-proteinuric, isolated proteinuria and PET groups was 36 weeks, IQR 35–37 weeks; 36 weeks, IQR 35–36 and 35 weeks, IQR 33–36 respectively, p = 0.001 (Table 3). Rates of PTL prior to 34 weeks of gestation in these groups were 11.2%, 3% and 25.9% respectively, which was also statistically significant (p = 0.004) (Table 3). Rates of urgent cesarean sections were significantly higher in the PET group compared with the other two groups (40.7% vs 19.1% non-proteinuric group vs 20.9% isolated proteinuria group, p = 0.03).

The rate of composite adverse pregnancy outcome was higher in IHCP patients with PET compared -with patients with and without proteinuria (51.9%, 14/27 vs. 26.9%, 18/67 vs. 27.5%, 49/178, respectively, p = 0.03).

Neonatal outcomes including: FD, neonatal death, Apgar score 5 min <7 and NICU admission were all comparable between the three groups. Median birth weight was lower in the PET group compared with non-proteinuric and proteinuric groups. Of note, median arterial umbilical cord pH in the proteinuric and PET groups was lower compared with non-proteinuric group, and the median venous umbilical cord pH was lower in the PET group compared with non-proteinuric and proteinuric groups. Nevertheless, rates of arterial pH < 7.1 were comparable among groups (Table 4).

**Table 4:** Neonatal outcomes.

	Non-proteinuric (n = 216)	Proteinuric with normal BP (n = 88)	Preeclampsia (n = 40)	*p-*value
Birth weight (grams) ^c^^,^^d^	2209.5 (1888–2589)	2420 (2234.75–2815)	2180 (1660–2515)	**0.002**
Birth weight Percentiles ^c^^,^^d^	51 (28–69)	64 (48–82)	56 (17–76)	**0.001**
Fetal Death	2 (0.9)	0 (0)	0 (0)	0.551
Neonatal death	1 (0.5)	0 (0)	1 (2.5)	0.211
Apgar 1 min	9 (9–9)	9 (9–9)	9 (9–9)	0.135
Apgar 5 min	10 (10–10)	10 (10–10)	10 (10–10)	0.399
Apgar 5 min <7	3 (1.4)	2 (1.1)	2 (5%)	0.244
NICU admission	23 (10.6)	10 (11.4)	4 (10)	0.97
Umbilical artery pH ^b^^,^^**c**^	7.29 (7.26–7.31)	7.27 (7.21–7.3)	7.26 (7.22–7.3)	**0.012**
Umbilical artery pH<7.1	1 (0.9)	2 (3.8)	1 (3.2)	0.411
Umbilical vein pH ^**c**^^,^^d^	7.3 (7.27–7.34)	7.31 (7.27–7.35)	7.29 (7.24–7.3)	**0.025**

^a^ Data are presented as median and interquartile range (IQR) OR N (%).

BP- Blood pressure.

pH data available to 113, 53 and 31 neonates from the non-proteinuric, proteinuric and preeclampsia groups respectively.

b—P value < 0.05 in a comparison between non-proteinuric and Proteinuric with normal BP.

c- P value < 0.05 in a comparison between non-proteinuric and pre-eclampsia.

d- P value < 0.05 in a comparison between proteinuric with normal BP and pre-eclampsia.

The median time between the onset of IHCP symptoms and diagnosis of PET was 3 weeks.

In our cohort, there were two cases of FD, both in twin pregnancies. The first case was FD of both fetuses at 27 weeks of gestation in monochorionic diamniotic pregnancy which was complicated with amniotic fluid discordancy and subsequent disruption of the membranes following amnioreduction. The second case was FD of a co-twin with growth-retardation and reversed flow at the umbilical artery, in dichorionic diamniotic pregnancy. Fetal death was diagnosed at 31 weeks of gestation and this patient was diagnosed with moderate IHCP and had PPROM one week before the IHCP was diagnosed.

## Discussion

The results of this study demonstrate that while 34.5% of patients with cholestasis had proteinuria, only 28.7% (27/94) of the proteinuria group had elevated blood pressure and met the criteria of PET. Thus, approximately one of every three patients with IHCP will have proteinuria, suggesting that this may be a common and heretofore unreported clinical characteristic of IHCP. In addition, the presence of PET rather than proteinuria *per se* may be associated with adverse pregnancy outcomes.

In the present study, 9.9% (27/272) of the patients with IHCP fulfilled the criteria of PET. This rate is higher than the prevalence reported in the general population (2–8%) [41]. Previous studies described a relationship between IHCP and PET [11,12,47,48], and a plausible hypothesis that elevated serum bile acids are a risk for PET had been suggested [11,49–53]. Common inflammatory and hypoxic processes may link these obstetric conditions [54–58]. Importantly, diagnosis of PET in the context of IHCP can be misleading as both conditions share similar laboratory abnormalities including elevated liver enzymes.

To our knowledge, the link between IHCP and proteinuria was not previously addressed. In our study, 24.6% of IHCP patients had proteinuria with normotensive pressure, rates that are higher than the reported rates (up to 8%) of isolated proteinuria in the pregnant population [17]. This extremely high rate of proteinuria raises the question whether proteinuria is a clinical feature of IHCP rather than part of “hidden” or “atypical” PET.

Despite the fact that the presence of proteinuria in IHCP may sound contra intuitive, there is a well-established biological plausibility for this association: 1. Chronic cholestatic liver diseases are associated with tubulointerstitial nephropathies [20–22]; 2. Accumulation of bile acids in the serum may contribute to endothelia injury in the kidney [52,59]; via oxidative stress [52] which promotes the formation of a of vasoactive mediators which in turn cause renal vasoconstriction and thus reducing the glomerular filtration rate [52]; 3. Abnormal biochemical functions of kidney glomeruli and tubules were more common in patients with IHCP [51]; 4. Two major receptors for bile acid have been identified in the kidney: the nuclear receptor farnesoid X receptor (FXR) and the membrane-bound, G protein-coupled bile acid receptor 1 (GPBAR1, TGR5) [60]. Recently, bile acids have emerged as important factors of renal pathophysiology by activating these receptors and transcription factors implicated in lipids and carbohydrates metabolism and in inflammation and renal fibrosis; and 5. There is increased prevalence of PET among patients with IHCP [11,12,47,61].

Raz et al. [11] reported a relationship between IHCP and PET. In their study PET usually presented 2–4 weeks after the diagnosis of IHCP. The findings of the present study are consistent with the abovementioned report as the median time between the onset of symptoms and diagnosis of PET was 3 weeks. Of note, this observation may suggest that TBA may induce a cascade of events that results in cholemic nephropathy and proteinuria as suggested by Raz et al.

Further research is needed, including prospective studies addressing the rates of proteinuria, as well as the concentrations of factors linked to PET and proteinuria, including soluble fms-like tyrosine kinase (sFlt-1), placental growth factor (PlGF), soluble endoglin (sEng), and free Vascular endothelial growth factor (VEGF) in order to identify the molecular and cellular mechanism(s) that may cause proteinuria in patients with IHCP [62–65].

Mroczko et al. [66–68] described an increase in the activity of total Alcohol Dehydrogenase (ADH) and ADH I isoenzyme in the serum of women with ICP, which seems to be caused by the release of this isoenzyme from damaged liver cells to the blood. Further research should investigate the correlation with ADH levels; which reflects tissue injury, and levels of proteinuria.

Our study had several strengths including a relatively large study group, with standardized management protocol and uniform clinical and laboratory evaluation. To the best of our knowledge, this is the first study that addresses the rate of proteinuria in patients with IHCP, as well as the first to determine the clinical significance of this finding in patient diagnosed with IHCP.

The limitations of our study should also be acknowledged. The retrospective design precludes comment regarding causation. In addition, the exclusion of patients who did not complete 24-hour urine collection may introduce a selection bias. Of note is that part of the patients excluded from this study due to the lack of 24-hour urine collection were patients presenting at term and thus an immediate induction was recommended. However, using protein spot samples may help overcoming these limitations. Moreover, no assessment for proteinuria was made early in pregnancy; therefore, we cannot determine if the proteinuria is de novo.

## Conclusions

Approximately 35% of patients with IHCP have proteinuria, suggesting that proteinuria might be a clinical feature of IHCP. The presence of PET rather than isolated proteinuria is associated with adverse pregnancy outcome. Proteinuria is regarded as an exclusive characteristic of PET and has not been reported in any other “great obstetrical syndrome”. Thus, the results of the present study are not only intriguing, but also might be of special clinical importance as the discrimination between PET and IHCP is a common clinical challenge with implications on patient management and neonatal outcome.
